# Supplementary figures and images for: Application of carbon dioxide to the skin and muscle oxygenation of human lower-limb muscle sites during cold water immersion
Source: PeerJ. 2020 Aug 21;8:e9785. doi: 10.7717/peerj.9785 (PMC7444506; doi:10.7717/peerj.9785)

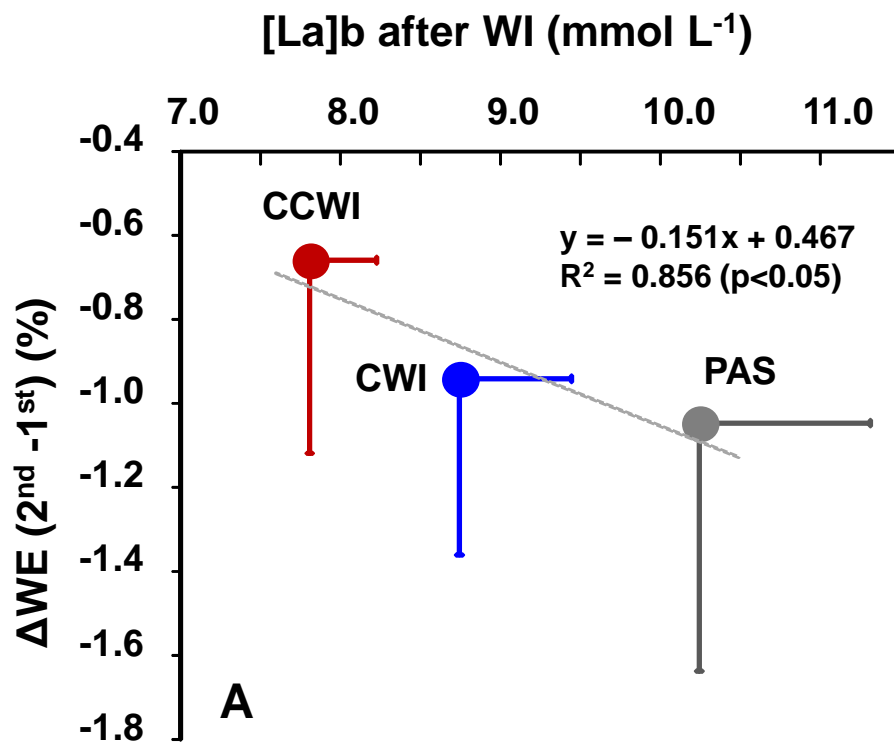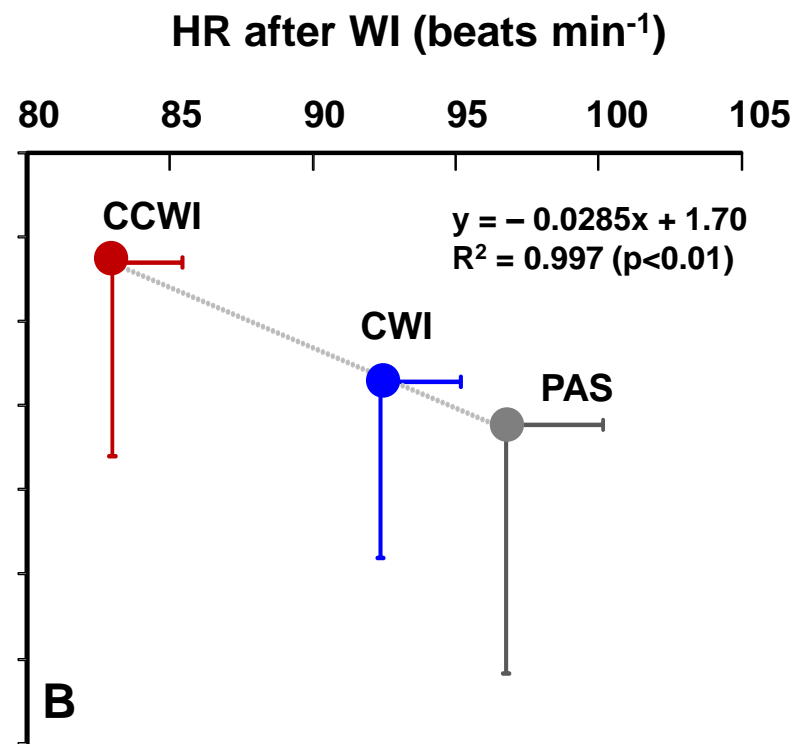

Supplement: Figure S1 — The relationship between the decrease in work efficiency (ΔWE) from the 1st to 2nd bouts and the [La]b (A) and HR (B) after each recovery intervention. Worsened WE at the 2nd bout was associated with both the [La]b and HR after the recovery interventions. [file peerj-08-9785-s001.pdf]
